# Supplementary material for: Hand2 inhibits kidney specification while promoting vein formation within the posterior mesoderm
Source: eLife. 2016 Nov 2;5:e19941. doi: 10.7554/eLife.19941 (PMC5132343; doi:10.7554/eLife.19941)
Supplement: Figure 2—source data 1. — The number of Pax2a+ cells was quantified on the indicated dates of analysis. For wild-type and hans6 embryos, representative 250 μm long regions of IM were analyzed, while for hs:hand2 embryos, 500 μm long regions of IM were analyzed. For hs:hand2 embryos, the IM on both the left and right sides of the embryo were analyzed independently when the dissection and preparation of the sample allowed. All values were normalized to represent the number of cells per 100 μm. Average number of Pax2a+ cells per 100 μm and standard deviation are represented in Figure 2G. DOI: http://dx.doi.org/10.7554/eLife.19941.007 [file elife-19941-fig2-data1.docx]

**Pax2a^+^ Cells in Wild-type, *han^s6^*, and *hs:hand2* Intermediate Mesoderm**

| Date/embryo | Genotype | Pax2a^+^ |
| --- | --- | --- |
| 12/13/15 |  | **Per 250um** |
| 1 | Wild-type | 72 |
| 3 | *han^s6^* | 132 |
|  |  |  |
| 1/3-4/16 |  | **Per 250um** |
| 1 | Wild-type | 82 |
| 2 | *han^s6^* | 154 |
| 5 | Wild-type | 83 |
| 7 | Wild-type | 84 |
| 8 | *han^s6^* | 123 |
| 9 | Wild-type | 103 |
| 10 | *han^s6^* | 163 |
| 11 | *han^s6^* | 158 |
| 12 | Wild-type | 99 |
| 13 | Wild-type | 90 |
| 15 | *han^s6^* | 152 |
| 16 | Wild-type | 119 |
| 17 | Wild-type | 97 |
| 18 | Wild-type | 78 |
|  |  |  |
| 1/10/16 |  | **Per 250um** |
| 1 | *han^s6^* | 118 |
| 2 | *han^s6^* | 154 |
| 3 | Wild-type | 74 |
| 4 | Wild-type | 87 |
| 5 | Wild-type | 76 |
| 6 | *han^s6^* | 98 |
| 8 | *han^s6^* | 105 |
|  |  |  |
| 9/13/16 |  | **Per 500um** |
| 1 (side1) | *hs:hand2* | 34 |
| 1 (side2) | *hs:hand2* | 7 |
| 2 (side1) | *hs:hand2* | 39 |
| 2 (side2) | *hs:hand2* | 26 |
| 3 (side1) | *hs:hand2* | 28 |
| 3 (side2) | *hs:hand2* | 35 |
| 4 (side1) | *hs:hand2* | 53 |
| 4 (side2) | *hs:hand2* | 28 |
| 5 (side1) | *hs:hand2* | 13 |
| 5 (side2) | *hs:hand2* | 106 |
| 6 (side1) | *hs:hand2* | 109 |
| 7 (side 1) | *hs:hand2* | 53 |
| 7 (side 2) | *hs:hand2* | 80 |
| 8 (side 1) | *hs:hand2* | 0 |
| 8 (side 2) | *hs:hand2* | 0 |
| 9 (side 1) | *hs:hand2* | 0 |
| 9 (side 2) | *hs:hand2* | 0 |
| 9 (side 1) | *hs:hand2* | 0 |
| 9 (side 2) | *hs:hand2* | 0 |

| Genotype (n) | Pax2a^+^ per 250um |
| --- | --- |
| Wild-type (n=13) | 88 + 13.4 |
| *han^s6^* (n=10) | 135.7 + 22.4 |

| Genotype (n) | Pax2a^+^ per 500um |
| --- | --- |
| *hs:hand2* (n=19) | 32 + 34.9 |

| Genotype (n) | Pax2a^+^ per 100um | p-value (compared to wild-type) |
| --- | --- | --- |
| Wild-type (n=13) | 35.2 + 5.4 |  |
| *han^s6^* (n=10) | 54.3 + 9.0 | p < 0.0001 |
| *hs:hand2* (n=19) | 6.4 + 7.0 | p < 0.0001 |
