# Supplementary material for: Hand2 inhibits kidney specification while promoting vein formation within the posterior mesoderm
Source: eLife. 2016 Nov 2;5:e19941. doi: 10.7554/eLife.19941 (PMC5132343; doi:10.7554/eLife.19941)
Supplement: Figure 2—figure supplement 2—source data 1. — The numbers of Pax2a+ cells and Pax2a+ pH3+ cells were quantified on the indicated dates of analysis. 400 μm long regions of IM were analyzed, and values were normalized to represent the number of cells per 100 μm. A proliferation index (percentage of Pax2a+ pH3+ cells per Pax2a+ cells) was calculated. Average numbers with standard deviation and proliferation index are represented in Figure 2—figure supplement 2C. DOI: http://dx.doi.org/10.7554/eLife.19941.010 [file elife-19941-fig2-figsupp2-data1.docx]

**Pax2a^+^ pH3^+^ Cells in Wild-type and *han^s6^* Intermediate Mesoderm**

| Date/embryo | Genotype | Pax2a^+^ pH3^+^ | Pax2a^+^ pH3^+^ | Pax2a^+^ | Pax2a^+^ | (Pax2a^+^ pH3^+^/ Pax2a^+^) x 100 |
| --- | --- | --- | --- | --- | --- | --- |
| 9/5/16 |  | **Per 400um** | **Per 100um** | **Per 400um** | **Per 100um** |  |
| 1 | Wild-type | 4 | 1 | 157 | 39.25 | 2.5 |
| 2 | *han^s6^* | 6 | 1.5 | 199 | 49.75 | 3.0 |
| 3 | Wild-type | 3 | .75 | 134 | 33.5 | 2.2 |
| 4 | *han^s6^* | 6 | 1.5 | 209 | 52.25 | 2.9 |
| 5 | Wild-type | 5 | 1.25 | 152 | 38 | 3.3 |
| 6 | Wild-type | 4 | 1 | 103 | 25.75 | 3.9 |
| 7 | *han^s6^* | 3 | .75 | 183 | 45.75 | 1.6 |
| 8 | *han^s6^* | 6 | 1.5 | 193 | 48.25 | 3.1 |
| 9 | *han^s6^* | 3 | .75 | 227 | 56.75 | 1.3 |
| 10 | Wild-type | 2 | .5 | 133 | 33.25 | 1.5 |
| 11 | Wild-type | 2 | .5 | 142 | 35.5 | 1.4 |
| 13 | Wild-type | 2 | .5 | 168 | 42 | 1.2 |
| 14 | Wild-type | 3 | .75 | 105 | 26.25 | 2.9 |
|  |  |  |  |  |  |  |
| 8/25/15 |  |  |  |  |  |  |
| 1 | Wild-type | 4 | 1 | 129 | 32.25 | 3.1 |
| 2 | *han^s6^* | 6 | 1.5 | 244 | 61 | 2.5 |
| 3 | *han^s6^* | 1 | .25 | 206 | 51.5 | 0.5 |
| 4 | Wild-type | 1 | .25 | 144 | 36 | 0.7 |
| 6 | Wild-type | 5 | 1.25 | 181 | 45.25 | 2.8 |
| 7 | Wild-type | 4 | 1 | 135 | 33.75 | 3.0 |
| 8 | *han^s6^* | 4 | 1 | 167 | 41.75 | 2.4 |
| 9 | Wild-type | 4 | 1 | 118 | 29.5 | 3.4 |
| 10 | Wild-type | 4 | 1 | 164 | 41 | 2.4 |
