# Supplementary material for: Hand2 inhibits kidney specification while promoting vein formation within the posterior mesoderm
Source: eLife. 2016 Nov 2;5:e19941. doi: 10.7554/eLife.19941 (PMC5132343; doi:10.7554/eLife.19941)
Supplement: Figure 7—source data 1. — The numbers of GFP+ and Pax2a+ GFP+ cells were quantified on the indicated dates of analysis. Representative 250 μm long regions of IM were analyzed, and values were normalized to represent the number of cells per 100 μm. Average numbers and standard deviation are represented in Figure 7E. DOI: http://dx.doi.org/10.7554/eLife.19941.017 [file elife-19941-fig7-data1.docx]

**GFP^+^ and Pax2a^+^ GFP^+^ Cells in Wild-type and *han^s6^* Intermediate Mesoderm**

Per 250um

| **Date/embryo** | **Genotype** | **GFP^+^** | **Pax2a^+^ GFP^+^** |
| --- | --- | --- | --- |
| 12/13/15 |  |  |  |
| 1 | Wild-type | 56 | 2 |
| 3 | *han^s6^* | 50 | 24 |
|  |  |  |  |
| 1/3-4/16 |  |  |  |
| 1 | Wild-type | 45 | 6 |
| 2 | *han^s6^* | 49 | 30 |
| 5 | Wild-type | 52 | 2 |
| 7 | Wild-type | 47 | 4 |
| 8 | *han^s6^* | 33 | 31 |
| 9 | Wild-type | 46 | 3 |
| 10 | *han^s6^* | 41 | 39 |
| 11 | *han^s6^* | 44 | 22 |
| 12 | Wild-type | 72 | 2 |
| 13 | Wild-type | 60 | 3 |
| 15 | *han^s6^* | 59 | 29 |
| 16 | Wild-type | 77 | 3 |
| 17 | Wild-type | 68 | 5 |
| 18 | Wild-type | 62 | 2 |
|  |  |  |  |
| 1/10/16 |  |  |  |
| 1 | *han^s6^* | 44 | 31 |
| 2 | *han^s6^* | 43 | 32 |
| 3 | Wild-type | 41 | 5 |
| 4 | Wild-type | 55 | 3 |
| 5 | Wild-type | 55 | 5 |
| 6 | *han^s6^* | 43 | 37 |
| 8 | *han^s6^* | 60 | 27 |

Per 250um

| **Genotype (n)** | **GFP+** | **Pax2a^+^ GFP^+^** |
| --- | --- | --- |
| Wt (n=13) | 56.6 + 10.9 | 3.5 + 1.4 |
| *han^s6^* (n=10) | 46.6 + 8.2 | 30.2 + 5.2 |

Per 100um

| **Genotype (n)** | **GFP+** | **Pax2a^+^ GFP^+^** |
| --- | --- | --- |
| Wt (n=13) | 22.6 + 4.4 | 1.4 + 0.6 |
| *han^s6^* (n=10) | 18.6 + 3.3 | 12.1 + 2.1 |
| p-value | 0.0259 | < 0.0001 |
